# 1-km resolution rebound surfaces and paleotopography of glaciated North America since the Last Glacial Maximum

**DOI:** 10.1038/s41597-023-02566-5

**Published:** 2023-10-23

**Authors:** Pierre-Marc Godbout, Etienne Brouard, Martin Roy

**Affiliations:** 1 Geological Survey of Canada, Natural Resources Canada, 601 Booth Street, Ottawa, ON K1A 0E8 Canada; 2 Department of Earth and Atmospheric Sciences & GEOTOP Research Center, University of Quebec at Montreal, C.P. 8888, Succ. Centre-ville, Montreal, QC H3C 3P8 Canada

**Keywords:** Geology, Geography, Geomorphology

## Abstract

We present a series of 1-km spatial resolution rebound (isobase) surfaces based on publicly distributed predictions obtained from the glacio-isostatic adjustment models known as ICE-5G (VM2 L90), ICE-6G_C (VM5a) and ICE-7G_NA (VM7). Our objective is to provide readily accessible tools for a broad range of geological and paleoenvironmental studies, and to facilitate direct comparison between models’ predictions and field-based observations. Rebound surfaces were interpolated at the scale of North American ice sheets (35.5°-89.5°N; 45°-165°W) and for each time increment of the models (1,000-500 yrs, between 26,000-21,000 yrs BP and present-day). The assessment of the interpolations indicates that the rebound surfaces have an overall vertical accuracy of ∼0.4 m compared to original ICE-xG outputs. These rebound surfaces were combined with the GEBCO 2021 present-day elevation grid to reconstruct the paleotopography for each time increment of the models and are all presented as raster files that can be easily integrated into geographical information systems. The resulting datasets therefore provide a unique support for geological, paleoenvironmental and archeological studies.

## Background & Summary

Our understanding of the evolution of the Earth system generally relies on observational data that provide basic boundary conditions for the development of complex numerical models. The ability of these models to faithfully reproduce past and current changes in the different components of the Earth system ultimately allows for simulations of future changes. As such, glacial isostatic adjustment (GIA) models represent unique tools to gain unparalleled insights about the intricate interactions between the cryosphere, the solid Earth and climate, as demonstrated by detailed analysis of past changes in relative sea level (RSL) and in the projections of the anticipated changes to come (e.g., refs. ^1,2^). However, the predictions of these models have yet to be fully integrated into empirical research due to the gap that exists between the modelling and observational (field-based) studies. Indeed, the geological (observational) and GIA modelling communities often operate separately, which can inhibit full-scale analyses of field-based datasets, lead to incomplete interpretations, and thus introduce some uncertainty into the models. This lack of synergy could be explained in part by model outputs that are commonly provided in formats that are often difficult to integrate into geoscience studies, while presenting a spatial resolution that is often too low to be used in paleotopographic reconstructions that generally derive from finer-scale field-based investigations. The development of easily accessible formats of model outputs would facilitate direct comparisons with geological data and lead to a better assessment of model performance. At the same time, such outputs should provide specific targets for geoscientists to collect data and thus generate new constraints with an increased precision that would in turn result in more robust boundary conditions for models.

In the last decades, significant advances in the understanding of the history of global sea-level variations linked to the growth and decay of the North American ice sheets during the last glacial-deglacial cycle have been made by GIA inversion-based numerical models integrating ice thickness/loading history and Earth’s radial viscosity models – such as the series of ICE-xG (VMy) models: ICE-3G (VM1)^3^, ICE-4G (VM2)^4,5^, ICE-5G (VM2)^6,7^, ICE_6G_C and D (VM5a)^8–10^ and ICE-7G_NA (VM7)^11–13^. Other relevant contributions with an equally good fit to observations also include the predictions generated by different combinations of Earth-ice models and parametrization approaches known as the ANU LW-6 (E-6)^14,15^, GLAC-1D^16^, Laur16^17^ and NAICE models^18^. Here we focus on the ICE-xG (VMy) since these models are extensively validated, in addition to be constantly improved (or refined) to fit new global field-based and observational constraints. Applications of the ICE-xG (VMy) predictions are numerous and include past/present-day ice-sheet^19^ and paleoclimate modelling^20^, transient simulations of the last deglaciation^21^, simulations of RSL changes and drainage pathways during critical time intervals of the last deglaciation^22,23^, paleotopographic reconstructions of Beringia^24^, governing parameters of ice stream dynamics (e.g., ref. ^25^), reconstructions of glacial lakes^26–28^, calibration of ^10^Be production sites and corrections of surface exposure ages (e.g., ref. ^29^), evaluation of North American plate angular velocity^30^, and evaluation of the response of hydrocarbon reservoirs to the GIA-induced vertical motion^31^. One of the key predictions made by the combination of ICE-xG (VMy) models consists in the postglacial rebound (PGR) component of the GIA – i.e., the viscoelastic deformation of the Earth’s surface in response to variations in surface loading generated by ice sheets growth and decay (e.g., ref. ^32^), which causes the crust to rebound in regions formerly covered by or adjacent to ice sheets, and subside beneath ocean basins. In North America, the observed PGR is mainly the result of the Laurentide Ice Sheet (LIS) deglaciation after it reached its maximum thickness and extent at the Last Glacial Maximum (LGM; 26.5-19 ka)^33^. Information about the present-day uplift rates and their spatial distribution are mostly provided by Global Positioning System (GPS) stations, while past loading histories close to the former ice dispersal centers (domes) are inferred using relative sea-level curves in coastal regions (e.g., ref. ^34^) and by deformation (tilt) of former glacial lake strandlines further inland (e.g., refs. ^12,35^). However, the scarcity of direct geomorphological indicators of crustal deformation at the continental scale, combined with the low number and varying resolution of geochronological constraints in the core regions of the LIS limit the inference of accurate PGR patterns (i.e., isobases).

A common challenge to all GIA models like the ICE-xG (VMy) resides in the integration of their predictions in a Geographic Information System (GIS) to facilitate paleotopographic reconstructions at a much higher resolution than their native degree-scale resolution. In fact, the development of such reconstructions primarily resides in the capacity of extracting absolute values for the time-dependent evolution of the depth of crustal depression beneath the ice masses, i.e., the subglacial paleotopography (cf. true paleotopography of ref. ^4^), which requires the interpolation of continuous (isobase) raster grid surfaces. Such high-resolution rebound surfaces combined with accurate elevation data and ice-margin histories have the potential to significantly improve paleotopographic reconstructions, which form key elements in understanding the role of meltwater released by the changes in geometry of the LIS in climate perturbations of the last deglaciation (e.g., refs. ^23,36,37^).

Here, we derive 1-km resolution interpolated rebound (isobase) surfaces along with ice-free paleotopographic reconstructions of the glaciated North America since the LGM, based on the depth of the land deformation predicted by the three most recent iterations of the ICE-xG (VMy) models. The outputs include the predictions made by the ICE-7G_NA (VM7)^11–13^ model as well as those generated by the previous – but still relevant – ICE_6G_C (VM5a)^8,9^ and ICE-5G (VM2)^6,7^ versions, which have an equally or better fit to data in some regions. The main objective of this reconstruction is to provide accessible tools for geoscientists working in a wide array of fields (e.g., mapping, geomorphology, archeology, and geochronology) and to allow direct comparisons between the models’ predictions and the geological observations constraining these models^38,39^.

## Methods

### Data and GIS integration

The ICE-5G (VM2 L90), ICE-6G_C (VM5a) and ICE-7G_NA (VM7) datasets were downloaded from www.atmosp.physics.utoronto.ca/~peltier/data.php (last accessed 2022-12-19) in their native NetCDF format at a 1 × 1 degree global grid and were imported in ArcMap using the Make NetCDF Feature layer function. The layers were then converted to points shapefiles, each comprising the models’ predicted variables provided in the NetCDF files. Since different reference frame definitions were used through the evolution of the ICE-xG models^9,40,41^, and as they might not be supported in common GIS software’s, all the datasets were referenced in ArcMap using the World Geodetic System 1984 (WGS84) horizontal reference frame. For the reconstructions of rebound (isobase) surfaces, the vertical datum (or reference geoid surface) was kept unspecified as the land deformation computed for each time increment is the difference in elevation between two surfaces relative to the same vertical reference frame, therefore providing absolute deformation values (or difference in orthometric heights) in meters. Before proceeding with the interpolation process at the scale of the North American continent, all data points were projected using a common projection (i.e., Canada Albers Equal Area Conic), which is appropriate to preserve both area and length, and minimize distortion when interpolating continuous surfaces with evenly distributed point-data (e.g., ref. ^42^).

**Fig. 1:**
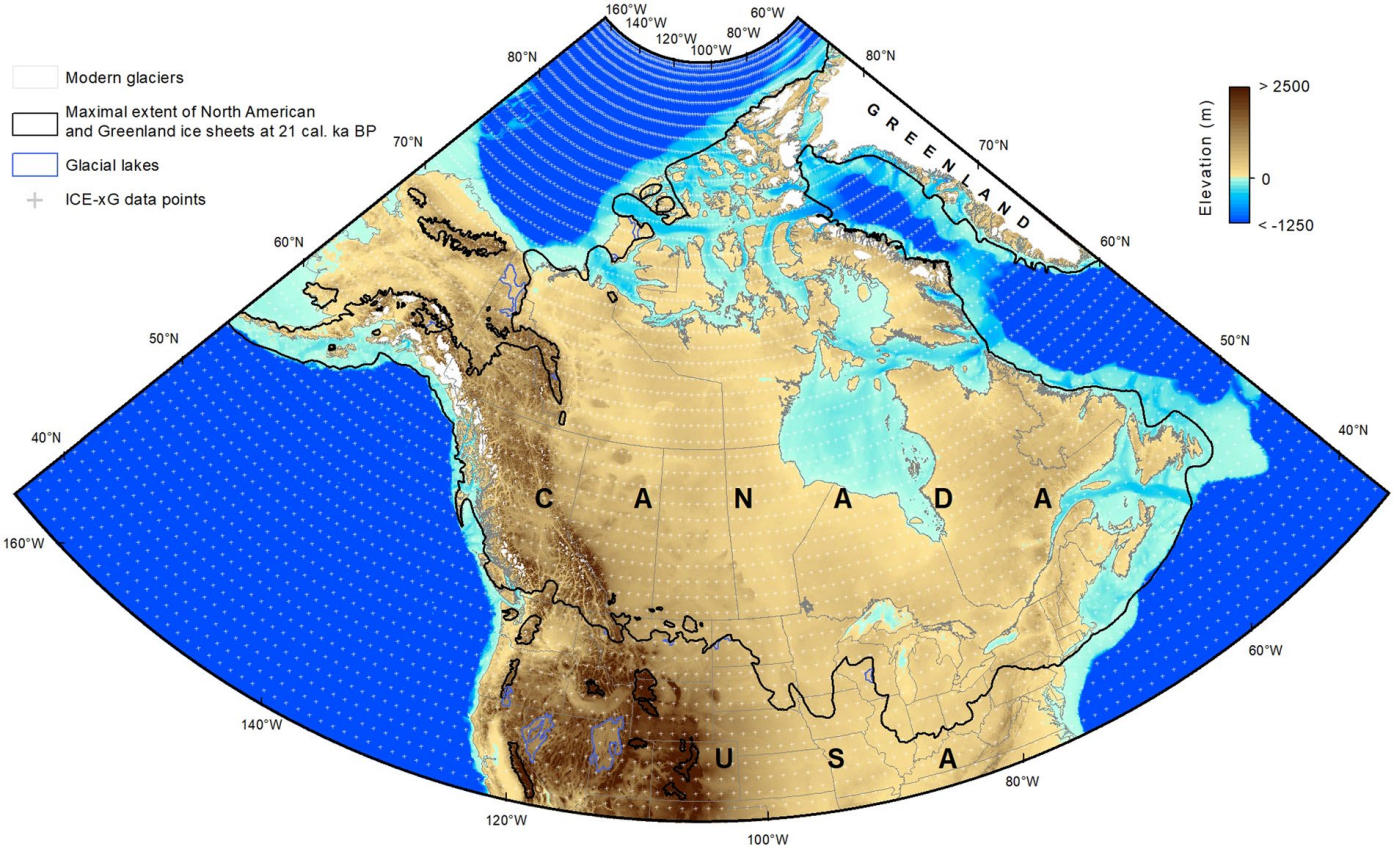
Extent of the reconstructions and data used therein. Extent of the ice sheets and glacial lakes at LGM (21 ka) are from refs. ^43,44^; present-day elevations and bathymetry are from the GEBCO 2021 grid^45^; and present-day glaciers are from the CanVec series (scale 1:5,000,000). The ICE-xG 1 × 1 degree point-data grid were selected between 35.5°-89.5°N and 45°-165°W, where a degree of latitude has a constant length of ∼111 km and a degree of longitude has a length varying between ∼91 km at 35.5°N, ∼52 km at 62.5°N and ∼1 km at 89.5°N.

For each time increment of the models, ∼6,600 points (GDB *Data_points*; see the Data Records section) were selected between 35.5°-89.5°N and 45°-165°W (Fig. 1) to compute the 1-km resolution ice-free paleotopography (see the Paleotopographic reconstructions section) at the scale of the LIS as predicted by the ICE-xG (VMy) models.

### ICE-5G (VM2 L90) version 1.2 land deformation

The ICE-5G (VM2 L90) dataset (hereafter referred to as ICE-5G) is composed of 3 variables: the surface altitude/orography (*orog*), the ice thickness (*sftgit*), and the ice mask (*sftgif*), the latter corresponding to the LIS, Cordilleran Ice Sheet (CIS) and Innuitian Ice Sheet (IIS) extent since LGM^43,44^. The first step to compute the land deformation of the land surface at X ka is to define the present-day (ice-free) topography, which will be used as the reference surface. The present-day topography is obtained by subtracting the ice thickness values at 0 ka from the orography at 0 ka. The same step is then repeated for each time increment of the model, i.e., by subtracting the ice thickness at X ka from the orography at X ka, yielding the subglacial paleotopography at X ka. The land deformation corresponds to the difference between the present-day (ice-free) topography at 0 ka and the (ice-free) paleotopography at X ka.

### ICE-6G_C (VM5a) land deformation

The ICE-6G_C (VM5a) dataset (hereafter referred to as ICE-6G) contains the same variables as the ICE-5G model, i.e., ice thickness (*stgit*) and orography (*orog*), but also contains the topography, which includes the bathymetry and the ice thickness (*Topo*), the topography difference from present (*Topo_Diff*), the land area fraction (*sftgif*) and ice area fraction (*sftlf)*, the latter two again corresponding to the ice sheet extents depicted in the Dyke *et al*. (refs. ^43,44^) reconstruction of the last deglaciation. The calculation of the land depression from the ICE-6G model is slightly more complex as the model is tuned to predict the presence of ice shelves that needs to be accounted for prior to the calculation. Where the ice margin is floating, the *orog* variable, as for the continental ice masses, gives the ice sheet surface elevation whereas the *Topo* variable corresponds to the ocean depth. Then, by selecting points where *orog* > 0 and the *Topo* ≤ 0, ice-shelves can be singled out. In this case, the surface loading is associated with the thickness of the water column below the floating ice mass instead of being associated with the ice mass itself. Therefore, under ice-shelves, the ice-free topography is directly given by the *Topo* variable at either 0 ka or X ka. Otherwise, for grounded continental ice masses, the ice-free topography at 0 ka or X ka is calculated by subtracting the ice thickness (*stgit*) from the topography (*Topo*). The land deformation corresponds to the difference between the grounded ice/ice-shelves free topography at 0 ka and the grounded ice/ice-shelves free topography at X ka, which is equivalent to the *Topo_Diff* variable for the non-glaciated/deglaciated areas.

### ICE-7G_NA (VM7) land deformation

Despite that the ICE-7G_NA (VM7) model (hereafter referred to as ICE-7G) represents the most recent and up-to-date iteration of the models, the computation of the land deformation at the scale of glaciated North America since LGM is complicated by the lack of information on ice shelves in the ICE-7G dataset. The predicted variables consist in the ice thickness (*stgit*), the topography (*Topo*) and the topography difference from present (*Topo_Diff*), but the orography variable (*orog*) that was used to target the ice-shelves with the ICE-6G model is absent from the ICE-7G outputs. Since the geometry of the ice sheet margins employed in the ICE-7G is also based on the deglaciation isochrones from Dyke *et al*. (refs. ^43,44^), it is possible to obtain the information needed to identify the land-based vs. marine/glaciolacustrine-terminating ice masses. As for the ICE-6G model, where the ice is grounded (i.e., *Topo* > 0 and *stgit* > 0), the orography variable can be derived without difficulty as it is equal to the *Topo* variable. However, where the *Topo* < 0 and *stgit* > 0, and where the ice could be floating, we used the ICE-6G predictions to derive a ratio for the height of ice above the water surface vs. the ice thickness (i.e., *orog*/*stgit = *0.115) and combined the result with the ICE-7G predicted ice thicknesses to compute the orography. Then, using the same basic conditions as for the ICE-6G model (i.e., *orog* > 0 and *Topo* ≤ 0), we were able to reveal most of the ice shelves and ultimately compute the ice-free land deformation for all the points.

However, since the ICE-7G model is not specifically tuned for the presence of ice shelves, this resulted in the computation of anomalous deformation values at few isolated point-locations close to the ice margins. Despite numerous attempts, we were unable to algorithmically singled out and remove these points. In the end, given the general smoothness of interpolated isobase surfaces (i.e., limited terrain/slope complexity) and the robustness of the results obtained with the spline interpolator (see the Technical Validation section), we chose to resolve this issue by manually removing the problematic points prior to the interpolation of the surfaces.

### Interpolation

**Fig. 2:**
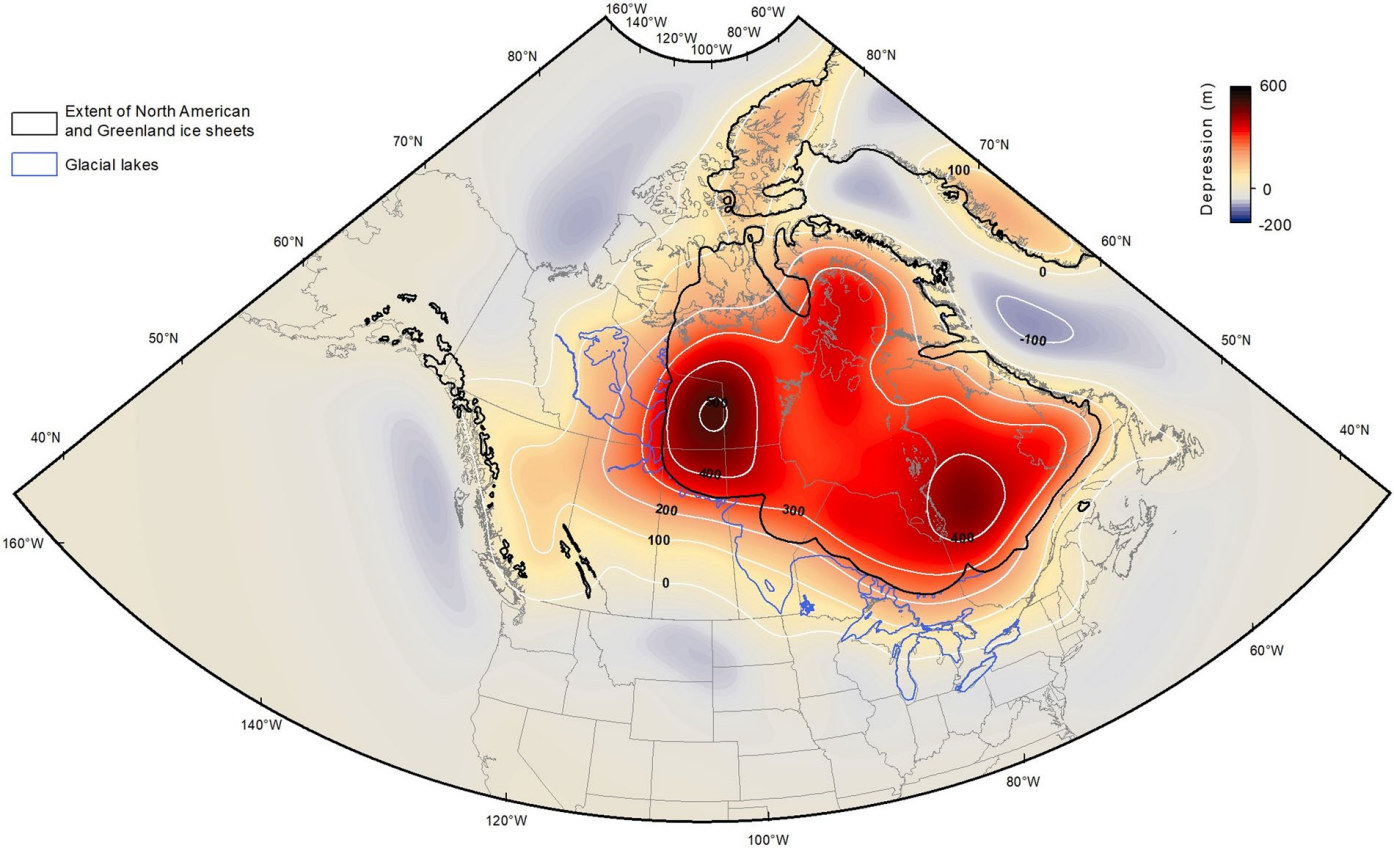
Example of a 1-km grid interpolated isobase surface. Isobase surface interpolated by spline and associated contours representing the depression of the land surface at 10 ka (ICE-6G) relative to the present-day sea level. Ice sheets and glacial lakes geometries at 10.2 cal ka BP (9 ^14^C ka BP)^43,44^.

A raster grid corresponding to the predicted isostatically-depressed (isobase) land surface was computed for each time increment. This was carried out in ArcMap using a spline algorithm to interpolate at a 1-km spatial resolution continuous surfaces between the projected 1 × 1 degree point-data grid. Other interpolation methods such as Natural Neighbours, Inverse Distance Weighted, Kriging and Topo To Raster were also tested. However, all were outperformed by the Spline algorithm, which is fitting a smooth surface with minimum curvature through the input points. Figure 2 shows an example of interpolated isobase surface obtained using the spline algorithm (ICE-6G; 10 ka).

### Paleotopographic reconstructions

**Fig. 3:**
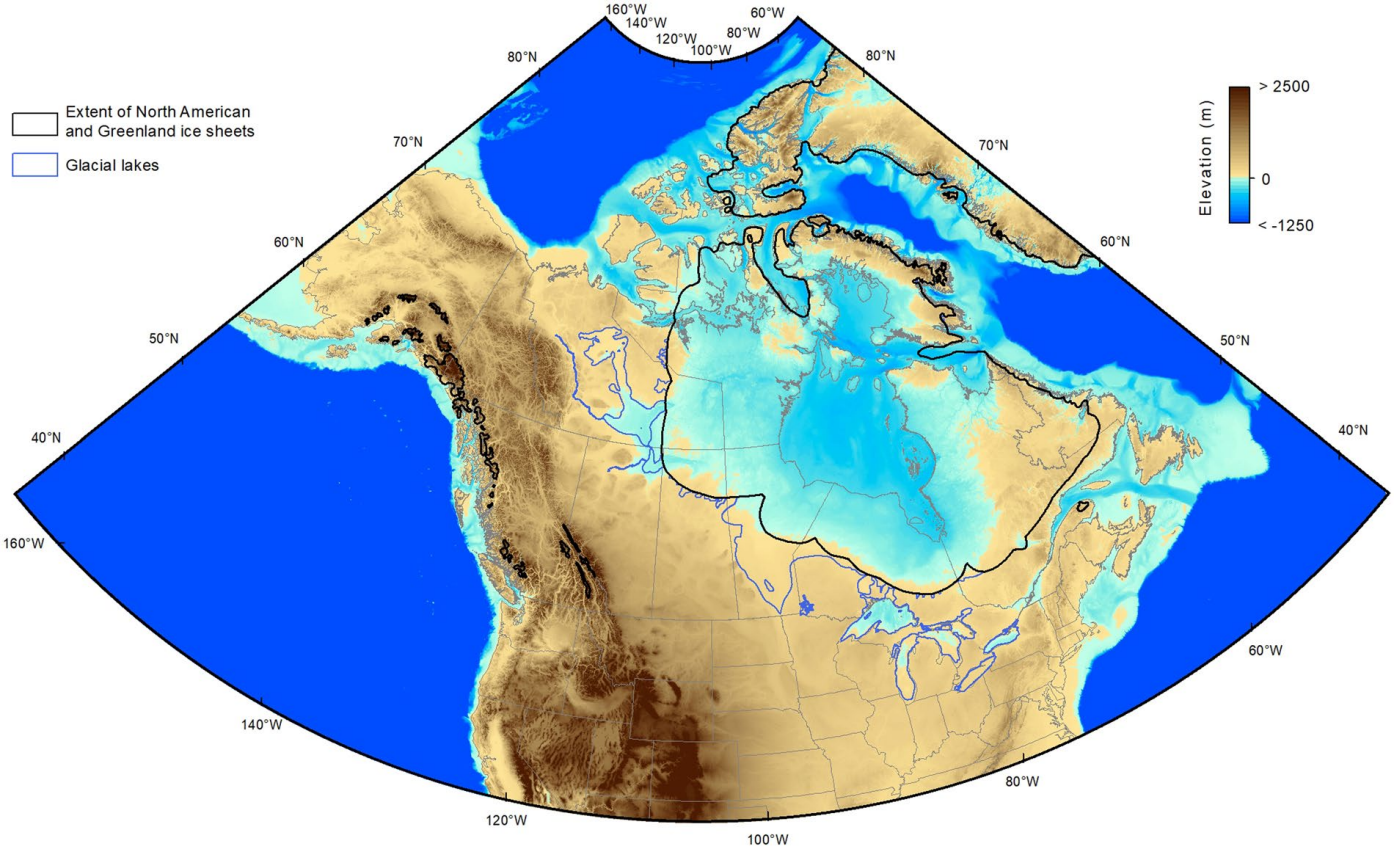
Example of a 1-km grid paleotopographic reconstruction. Paleotopographic reconstruction at 10 ka (ICE-6G) obtained by subtracting the isobase surface from the GEBCO present-day topography (including the bathymetry and the Greenland ice-free topography). The elevation is relative to the sea level at 10 ka. In coastal areas connected to the oceans and located beyond the extent of the ice sheets, the 0 m value corresponds to the predicted coastlines.

We used the GEBCO 2021 grid (ref. ^45^; hereafter referred to as GEBCO) to reconstruct the paleotopography and the paleobathymetry of the glaciated North America (Fig. 3). The GEBCO grid is a global terrain model updated from existing data (e.g., SRTM15 + V2.0; ref. ^46^) that offers a full coverage at a spatial resolution of 15 arc seconds (∼500 m pixel size at the equator) of the land and seafloor topography, as well as the Greenland sub-ice topography derived from the IceBridge BedMachine Greenland (Version 3; ref. ^47^). In the ocean, the GEBCO grid has an estimated absolute vertical accuracy (reported as root mean square error, RMSE) of 180 m nearshore and 150 m in the deep ocean^46^, while on land the absolute vertical accuracy is unspecified. The horizontal reference frame of the GEBCO is the same as the isobase surfaces (i.e., WGS84) and the elevations refer to geoidal heights provided by the Earth Gravitational Model 1996 (EGM96) reference frame. Since the isobase surfaces were interpolated at a 1-km spatial resolution, the GEBCO was resampled at the same grid size using bilinear interpolation before subtracting the isobase surface from the modern topography to reconstruct the paleotopography of glaciated North America (e.g., refs. ^48–51^). Consequently, the elevation of the paleo-digital elevation models (paleoDEMs) is referenced to the WGS84/EGM96 geoid.

## Data Records

Our methodological approach yielded a series of 132 rebound surfaces and associated paleoDEMs raster files (38 for the ICE-5G, 47 for the ICE-6G and 47 for the ICE-7G) deriving from interpolations at a 1-km spatial resolution for the area covered by the former North American ice sheets (i.e., between 35.5°-89.5°N and 45°-165°W), for time increments ranging from 500 to 1,000 yrs for the interval covering the LGM (26-21 ka BP) to present-day. The output files for each model are available on PANGAEA (10.1594/PANGAEA.947536; ref. ^52^) and are included in a set of geodatabases (GDBs; *Data_points*, *Contours*, *Deformation* and *Paleo_subice*). *Data_points* contain the original model variables converted from the NetCDF files for each time step (see Methods section for a complete listing of the variables) to which were appended the 0 ka variables (e.g., *Topo_0ka*, *stgit_0ka*) and the computed parameters such as the orography (*calc_orog*) and land deformation (*GIA*); *Contours* are 100 m contours of the interpolated land depression; *Deformation* are the interpolated land depression rasters; and *Paleo_subice* are the PaleoDEM rasters. The ICE-7G dataset also include the GDB *Data_points_used* which contains the files generated following the manual removal the problematic points (cf. *Data_points_all*) prior to the interpolation of the surfaces. No raster were generated for 0 ka since the deformation is equal to 0 for each model.

## Technical Validation

The best interpolation results were obtained through an iterative process using the regularized spline method (0 weight and 15 points) in ArcGIS. We assessed the overall vertical accuracy associated with this interpolator by selecting four time slices (5, 10, 15 and 20 ka) for each model (ICE-5G, -6G and -7G). The models’ predictions at each point location correspond to the reference (i.e., the checkpoints) against which the interpolated values must be compared to (e.g., ref. ^53^). This was accomplished by first extracting the interpolated values at each point location (n = 6,655 or less), then by computing the difference between the reference and the interpolated values to obtain the elevation errors (Tables 1, 2).

**Table 1:** Errors descriptive statistics for the 5 and 10 ka time slices.

		ICE-5G	ICE-6G	ICE-7G	ICE-7G*
5 ka	# of points	6,655	6,600	6,575	6,573
	Mean	0.00	0.00	0.00	0.00
	Median	0.00	0.00	0.00	0.00
	Min	−0.14	−0.18	−0.16	−0.15
	Max	0.14	0.18	0.18	0.14
	SD	0.03	0.03	0.02	0.02
	Q1	−0.01	−0.01	−0.01	−0.01
	Q3	0.01	0.01	0.01	0.01
	IQR	0.01	0.01	0.01	0.01
	Skewness	0.05	−0.12	−0.10	−0.13
	Kurtosis	4.40	6.90	7.23	6.70
	RMSE	0.03	0.03	0.02	0.02
	1.96 x RMSE	0.05	0.05	0.05	0.05
	95th perc.	0.06	0.06	0.05	0.04
10 ka	# of points	6,655	6,600	6,429	6,427
	Mean	0.00	0.00	0.00	0.00
	Median	0.00	0.00	0.00	0.00
	Min	−0.52	−0.51	−10.58	−2.32
	Max	0.54	0.53	1.40	1.19
	SD	0.09	0.09	0.16	0.09
	Q1	−0.02	−0.02	−0.02	−0.02
	Q3	0.02	0.02	0.02	0.02
	IQR	0.04	0.04	0.04	0.04
	Skewness	0.13	−0.17	−43.48	−2.09
	Kurtosis	5.17	5.48	2,825.50	71.44
	RMSE	0.09	0.09	0.16	0.09
	1.96 x RMSE	0.18	0.17	0.32	0.18
	95th perc.	0.22	0.20	0.20	0.14

The ICE-7G* corresponds to values calculated after removing the most extreme points (i.e., the min and the max) prior to statistic calculations. All values are in meters.

**Table 2:** Errors descriptive statistics for the 15 and 20 ka time slices.

		ICE-5G	ICE-6G	ICE-7G	ICE-7G*
15 ka	# of points	6,655	6,600	6,507	6,505
	Mean	0.00	0.00	0.00	0.00
	Median	0.00	0.00	0.00	0.00
	Min	−1.33	−0.80	−9.17	−4.74
	Max	1.28	0.73	0.59	0.56
	SD	0.16	0.14	0.19	0.15
	Q1	−0.03	−0.03	−0.03	−0.03
	Q3	0.03	0.03	0.03	0.03
	IQR	0.06	0.05	0.06	0.06
	Skewness	0.12	−0.30	−20.92	−5.32
	Kurtosis	6.81	4.10	963.46	164.60
	RMSE	0.16	0.14	0.19	0.15
	1.96 x RMSE	0.30	0.27	0.37	0.29
	95th perc.	0.36	0.33	0.32	0.23
20 ka	# of points	6,655	6,600	6,480	6,478
	Mean	0.00	0.00	0.00	0.00
	Median	0.00	0.00	0.00	0.00
	Min	−1.37	−0.86	−4.69	−0.77
	Max	1.22	0.74	2.65	0.61
	SD	0.16	0.14	0.15	0.14
	Q1	−0.03	−0.03	−0.03	−0.03
	Q3	0.03	0.03	0.03	0.03
	IQR	0.07	0.06	0.06	0.06
	Skewness	0.07	−0.27	−3.74	−0.29
	Kurtosis	6.51	3.91	146.11	3.42
	RMSE	0.16	0.14	0.15	0.14
	1.96 x RMSE	0.31	0.28	0.30	0.27
	95th perc.	0.36	0.33	0.33	0.24

The ICE-7G* corresponds to values calculated after removing the most extreme points (i.e., the min and the max) prior to statistic calculations. All values are in meters.

**Fig. 4:**
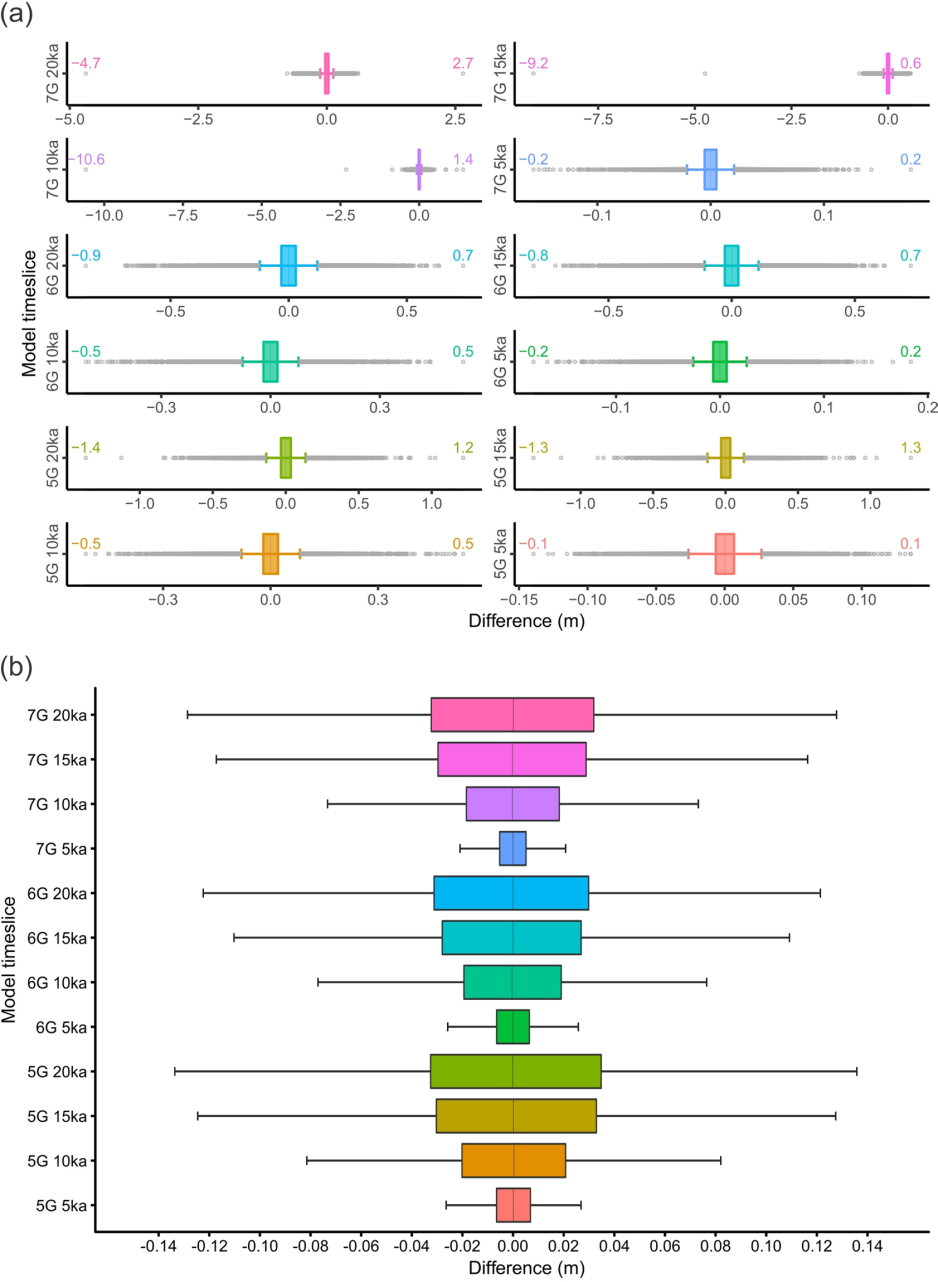
Boxplots showing the distribution of errors. The errors correspond to the difference between the interpolated values and the models’ predictions at each corresponding location. (**a**) With the outliers indicated; (**b**) Boxplot only. The upper and lower whiskers correspond to Q3 + 1.5 * IQR and Q1 - 1.5 * IQR, respectively^65,66^.

For the interpolations carried out using the ICE-5G and ICE-6G predictions, the descriptive statistics and the box plots computed for each time increment show distributions of errors that are concentrated and centered at the median (0 m), thus close to normal distributions with the mean error being around zero and the standard deviation (SD) and the RMSE being identical^53^. For all time slices, the error distributions are slightly right-skewed (positive) for the ICE-5G and left-skewed (negative) for the ICE-6G. For both models, the excess of kurtosis (>3) observed for all time slices reflects the presence of slightly heavier tails than observed with a normal distribution (Fig. 4a). For the ICE-7G predictions, the 5-ka box plot and the descriptive statistics are very similar to what is observed for all the time slices with the ICE-6G, i.e., approximately symmetric and normal distributions which are slightly left-skewed and heavy-tailed. However, the 10, 15 and 20 ka time slices reveal left-skewed and heavy-tailed distributions due to the presence of a few extreme outliers, probably due to the tuning of the model and/or generated by the greater number of points manually removed from the datasets (see the Methods section; Fig. 4a). The effect of these extreme values on the distributions is showed by the extreme values of kurtosis and skewness. A simple sensitivity test performed by computing all the descriptive statistics following the removal of the maximum and minimum values show a great improvement in both skewness and kurtosis, thereby highlighting the effect of those few extreme outliers on these parameters (ICE-7G*; Tables 1, 2).

Overall, for all the selected time slices, the distributions of errors are centered on 0 m (unbiased), symmetrical and approximating normal distributions. All three models show a greater dispersion of the data towards older time slices (Fig. 4b), which is likely due to the larger deformation and (slightly) more complex surface geometry (i.e., curvature) that the spline interpolator function needs to fit. The excess of skewness and kurtosis observed for all models limits the use of the RMSE to define the vertical accuracy of our reconstructions – an issue particularly important for 10, 15 and 20 ka time slices of the ICE-7G due to the presence of a few extreme outliers (Fig. 4a). Consequently, we report the overall accuracy of our interpolations using 95^th^ percentile error (Tables 1, 2) (e.g., refs. ^53,54^). For the ICE-5G model, these values correspond to 0.06 m (5 ka), 0.22 m (10 ka), 0.36 m (15 ka) and 0.36 m (20 ka). For the ICE-6G, these values are 0.06 m (5 ka), 0.20 m (10 ka), 0.33 m (15 ka) and 0.33 (20 ka) and for the ICE-7G model they correspond to 0.05 m (5 ka), 0.20 m (10 ka), 0.32 m (15 ka) and 0.33 (20 ka). The vertical accuracy of all the interpolated paleo-surfaces is therefore confidently determined at ∼0.4 m using both metrics.

Furthermore, the generalization of the datum used to reference each individual ICE-xG dataset in a GIS (i.e., WGS84) and the selection of the appropriate projection (i.e., Canada Albers Equal Area Conic) involve a transformation from the WGS84 to the NAD83 datum that results in coordinates differences of ∼1-2 m horizontally and ∼1 m vertically (e.g., refs. ^55,56^). While this ∼1 m must be added to the overall vertical uncertainties of ∼0.4 m resulting from the interpolation process (Fig. 2), the additional horizontal (positional) uncertainties generated by the use of the WGS84 reference frame are outweighed by the uncertainties associated with the resolution of the model (i.e., 1 × 1 degree) and the continental-scale (100-1,000’s km) of the modeled PGR. In this regard the data points can be considered as coincident and the ∼1-2 m horizontal error can be considered insignificant. Overall, the vertical accuracy associated with the projected/interpolated rebound surfaces from the ICE-5G, -6G and -7G predictions is estimated to be ∼1.4 m at the scale of the LIS. This outcome is again outweighed by the prevailing uncertainties inherent to the GIA models (e.g., ref. ^57^) and as such, each projected/interpolated rebound surfaces can be considered as exact with respect to the predictions.

## Usage Notes

All the 1-km resolution interpolated rebound surfaces produced in this study are available under the form of individual raster files (i.e., *Deformation* and *Paleo_subice* GDBs) that can be directly imported in ArcGIS. With routine raster operations, users will be able to modify the projection, adjust the spatial resolution and subtract the isobase raster grids from present-day high-resolution digital elevation models (DEMs; e.g., LiDAR-DEM) to rapidly generate high-resolution PaleoDEMs that can be used to support quantitative studies (e.g., ref. ^48^). The resampling of the isobase surfaces to higher (or lower) resolutions is not expected to significantly alter the overall ∼1.4 m vertical accuracy associated with the GIS referencing/projecting and interpolation process given the very low complexity level of the terrain involved (i.e., low and constant slopes/topographic gradients), in particular at the scale of the models native 1-degree resolution (e.g., ref. ^58^).

**Fig. 5:**
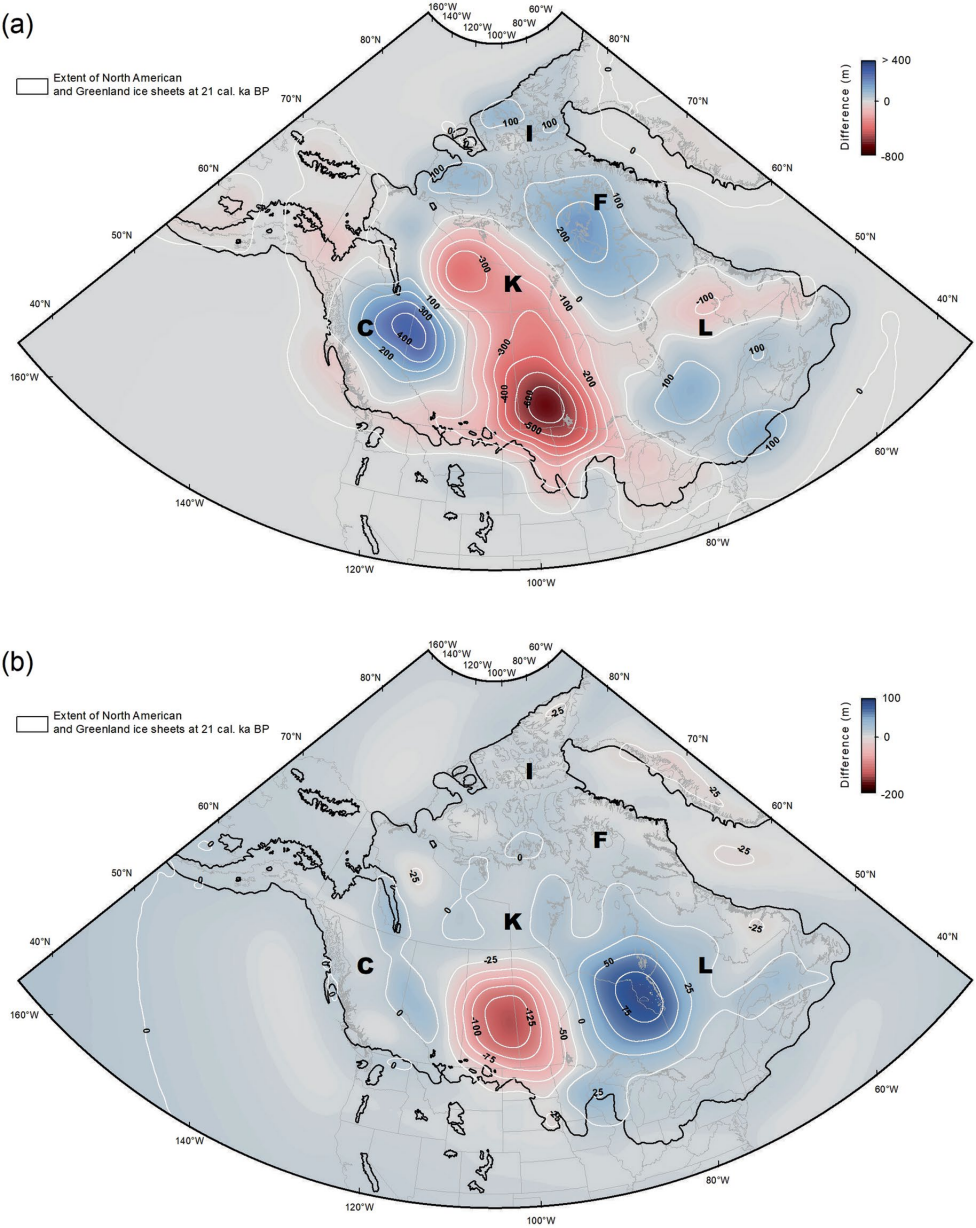
Differences in the models interpolated land deformation at LGM (21 ka). (**a**) Difference between the ICE-5G and ICE-6G and (**b**) the ICE-6G and ICE-7G models. The main regions of glaciated North America are denoted as: C = Cordilleran ice sheet, F = Foxe sector, I = Innuitian ice sheet, K = Keewatin sector and L = Labrador sector. Note the different min and max values of the scales in (a) and (b).

Figure 5 shows the main differences between the modelled land deformation between the ICE-5G/ICE-6G (a) and ICE-6G/ICE-7G (b) around the LGM (21 ka). These differences in topographic height between the different models respectively correspond to the redistribution of the ice loads presented in ref. ^11^ (cf. Figure 1b) and ref. ^12^ (cf. Fig. 15c), but with the amplitude of the Earth’s crust deformation being ∼1/3 of the ice thickness anomalies (between ± 1,000-2,000 m in some places). These differences between the models’ predictions highlight the improvements between each model iterations (beyond the viscosity profiles used), notably with the addition of space-geodetic constraints between the ICE-5G and ICE-6G, and the regional optimization of the model parameters between the ICE-6G and ICE-7G models. This has led to a more accurate prediction of the deformation along the border between the Alberta and the British Columbia and over the James Bay area, in addition to a significant reduction of the deformation in central Canada and in the Keewatin sector of the LIS, which are better aligned with the predictions made by other models such as the LAUR16^17,59^ and the NAICE^18^.

The resulting deformation is dominated by a double bull’s-eye pattern where the extrema straddles Hudson Bay – a pattern very similar to what is obtained from GRACE observations (cf. Fig. 6; ref. ^9^) or predicted by the ANU model^15,60^. Although there are major differences in ice loading histories between the ICE-5G and the ICE-7G models – which cause significant differences in terms of predicted land deformation in the core regions of the LIS –, the ICE-5G interpolated surfaces are still adequate in peripheral regions of the LIS (e.g., western Yukon and Canadian Arctic) and should still be considered to test the validity of the model against geological observations.

These differences between the models’ predictions clearly show the direct influence of the inferred ice-sheet loading history (i.e., the thickness of the ice sheet as a function of location and time) used to tune the models. Most of the ‘direct’ information documenting the temporal evolution of the ice thickness of the North American ice sheets derives from reservoir-corrected ^14^C RSL data that are primarily restricted to coastal regions, where the ICE-xG (VMy) models generally fit the observations within 2 sigma confidence limits (e.g., ref. ^9^). Inland, the observed present-day tilt of former glacial lake strandlines can provide this type of information, although at present, the scarcity of high-resolution records combined with the limited geochronological constraints increase uncertainties. The highest level of uncertainties occurs in the interior regions of the LIS (e.g., Keewatin sector) where ice margin geometries are poorly defined and the timing of ice retreat loosely pegged in the deglaciation framework, which translates into a significant decrease in the accuracy of any type of ice thickness reconstructions. Since the same ice extent history^43,44^ is used in the different ICE-xG models to infer ice thicknesses, it is advised that any intended paleotopographic reconstructions targeting regions where limited information exist minimally consider the uncertainty associated with the ice extent history - ultimately propagated in the computed land deformation -, by integrating the predictions of ± 2 time increments before 18 ka and to ± 1 time increment after 18 ka (e.g., refs. ^39,61^).

Accordingly, accounting for the uncertainty of the ice extent history provides a range of plausible ice margin configurations and associated land deformations for a given time interval. This permits paleotopographic reconstructions integrating different ice margin configurations (e.g., ref. ^61^) in association with predicted land deformations to evaluate key aspects of the LIS deglaciation such as the routing of the meltwaters. This can be achieved by flooding the (paleo)topography using GIS hydrology algorithms to better document the redistribution of meltwater between ice sheets and the ocean domain (e.g., refs. ^62,63^). Such methodology has been used effectively to facilitate correlation of scattered and discontinuous shorelines mapped using LiDAR-based DEMs in the eastern Lake Agassiz-Ojibway basin to establish lake stages^28^, and to evaluate the configuration the areal extent of Lake Agassiz-Ojibway prior to its final drainage^64^. These studies show how different sources of geological information comprised by ICE-xG prediction-based paleotopographic reconstructions can eventually contribute to produce realistic estimates of the meltwater volumes involved in certain drainage events of the last deglaciation, a key parameter in evaluating the impact of freshwater forcings that presumably triggered abrupt climate changes during the early Holocene.

## Data Availability

ESRI ArcGIS 10.6™ and later versions were used to process the data and produce the output datasets. No custom code has been used.

## References

[1] Alley RB, Clark PU, Huybrechts P, Joughin I (2005). Ice-sheet and sea-level changes. Science.

[2] Clark PU (2016). Consequences of twenty-first-century policy for multi-millennial climate and sea-level change. Nat. Clim. Change.

[3] Tushingham AM, Peltier WR (1991). Ice‐3G: A new global model of late Pleistocene deglaciation based upon geophysical predictions of post‐glacial relative sea level change. J. Geophys. Res.: Solid Earth.

[4] Peltier WR (1994). Ice age paleotopography. Science.

[5] Peltier WR (1996). Mantle viscosity and ice-age ice sheet topography. Science.

[6] Peltier WR (2002). Global glacial isostatic adjustment: Palaeogeodetic and space‐geodetic tests of the ICE‐4G (VM2) model. J. Quat. Sci.: Published for the Quaternary Research Association.

[7] Peltier WR (2004). Global glacial isostasy and the surface of the ice-age Earth: the ICE-5G (VM2) model and GRACE. Annu. Rev. Earth Planet. Sci..

[8] Argus DF, Peltier W, Drummond R, Moore AW (2014). The Antarctica component of postglacial rebound model ICE-6G_C (VM5a) based on GPS positioning, exposure age dating of ice thicknesses, and relative sea level histories. Geophys. J. Int..

[9] Peltier WR, Argus DF, Drummond R (2015). Space geodesy constrains ice age terminal deglaciation: The global ICE-6G_C (VM5a) model. J. Geophys. Res.: Solid Earth.

[10] Peltier WR, Argus DF, Drummond R (2018). Comment on “An assessment of the ICE‐6G_C (VM5a) glacial isostatic adjustment model” by Purcell et al. J. Geophys. Res.: Solid Earth.

[11] Roy K, Peltier WR (2015). Glacial isostatic adjustment, relative sea level history and mantle viscosity: reconciling relative sea level model predictions for the US East coast with geological constraints. Geophys. J. Int..

[12] Roy K, Peltier WR (2017). Space-geodetic and water level gauge constraints on continental uplift and tilting over North America: regional convergence of the ICE-6G_C (VM5a/VM6) models. Geophys. J. Int..

[13] Roy K, Peltier WR (2018). Relative sea level in the Western Mediterranean basin: a regional test of the ICE-7G_NA (VM7) model and a constraint on late Holocene Antarctic deglaciation. Quat. Sci. Rev..

[14] Lambeck K, Purcell A, Zhao J, Svensson NO (2010). The Scandinavian ice sheet: from MIS 4 to the end of the last glacial maximum. Boreas.

[15] Lambeck K, Purcell A, Zhao S (2017). The North American Late Wisconsin ice sheet and mantle viscosity from glacial rebound analyses. Quat. Sci. Rev..

[16] Tarasov L, Dyke AS, Neal RM, Peltier WR (2012). A data-calibrated distribution of deglacial chronologies for the North American ice complex from glaciological modeling. Earth Planet. Sci. Lett..

[17] Simon KM, James TS, Henton JA, Dyke AS (2016). A glacial isostatic adjustment model for the central and northern Laurentide Ice Sheet based on relative sea level and GPS measurements. Geophys. J. Int..

[18] Gowan EJ, Tregoning P, Purcell A, Montillet J-P, McClusky S (2016). A model of the western Laurentide Ice Sheet, using observations of glacial isostatic adjustment. Quat. Sci. Rev..

[19] Abe-Ouchi A (2015). Ice-sheet configuration in the CMIP5/PMIP3 Last Glacial Maximum experiments. Geosci. Model Dev..

[20] Matero IS, Gregoire LJ, Ivanovic RF (2020). Simulating the Early Holocene demise of the Laurentide Ice Sheet with BISICLES (public trunk revision 3298. Geosci. Model Dev..

[21] Kapsch ML, Mikolajewicz U, Ziemen F, Schannwell C (2022). Ocean response in transient simulations of the last deglaciation dominated by underlying ice‐sheet reconstruction and method of meltwater distribution. Geophys. Res. Lett..

[22] Pico T, Mitrovica J, Mix A (2020). Sea level fingerprinting of the Bering Strait flooding history detects the source of the Younger Dryas climate event. Sci. Adv.

[23] Tarasov L, Peltier WR (2005). Arctic freshwater forcing of the Younger Dryas cold reversal. Nature.

[24] Jakobsson M (2017). Post-glacial flooding of the Bering Land Bridge dated to 11 cal ka BP based on new geophysical and sediment records. Clim. Past.

[25] Velay‐Vitow J, Peltier WR, Stuhne GR (2020). The tides of the glacial ocean and their possible connection to Heinrich event instabilities of the laurentide ice sheet. J. Geophys. Res.: Oceans.

[26] Brown, V. H. *Ice stream dynamics and pro-glacial lake evolution along the north-western margin of the Laurentide Ice Sheet*. PhD Thesis. Durham University (2012).

[27] Hinck S, Gowan EJ, Lohmann G (2019). LakeCC: a tool for efficiently identifying lake basins with application to palaeogeographic reconstructions of North America. J. Quat. Sci..

[28] Godbout P-M, Roy M, Veillette JJ (2020). A detailed lake-level reconstruction shows evidence for two abrupt lake drawdowns in the late-stage history of the eastern Lake Agassiz-Ojibway basin. Quat. Sci. Rev..

[29] Jones R, Whitehouse P, Bentley M, Small D, Dalton A (2019). Impact of glacial isostatic adjustment on cosmogenic surface-exposure dating. Quat. Sci. Rev..

[30] Ding K, Freymueller JT, He P, Wang Q, Xu C (2019). Glacial isostatic adjustment, intraplate strain, and relative sea level changes in the eastern United States. J. Geophys. Res.: Solid Earth.

[31] MacDougall MDJ, Braun A, Fotopoulos G (2020). Implications of glacial isostatic adjustment on petroleum reservoirs in the Grand banks of Newfoundland. J. Geodyn..

[32] Whitehouse PL (2018). Glacial isostatic adjustment modelling: historical perspectives, recent advances, and future directions. Earth Surf. Dyn..

[33] Clark PU (2009). The last glacial maximum. Science.

[34] Simon KM (2014). A relative sea-level history for Arviat, Nunavut, and implications for Laurentide Ice Sheet thickness west of Hudson Bay. Quat. Res..

[35] Lewis, M., Breckenridge, A. & Teller, J. Reconstruction of isostatically-adjusted paleo-strandlines along the southern margin of the Laurentide Ice Sheet in the Great Lakes, Lake Agassiz and Champlain Sea basins. *Can. J. Earth Sci*. **59**, 10.1139/cjes-2021-0005 (2021).

[36] Broecker WS (1989). Routing of meltwater from the Laurentide Ice Sheet during the Younger Dryas cold episode. Nature.

[37] Condron A, Winsor P (2012). Meltwater routing and the Younger Dryas. Proc. Natl. Acad Sci. USA.

[38] Stokes CR (2015). On the reconstruction of palaeo-ice sheets: recent advances and future challenges. Quat. Sci. Rev..

[39] Yokoyama Y, Purcell A (2021). On the geophysical processes impacting palaeo-sea-level observations. Geosci. Lett..

[40] Argus DF, Peltier WR (2010). Constraining models of postglacial rebound using space geodesy: a detailed assessment of model ICE-5G (VM2) and its relatives. Geophys. J. Int..

[41] Argus DF (2010). The angular velocities of the plates and the velocity of Earth’s centre from space geodesy. Geophys. J. Int..

[42] Kuniansky EL (2017). Custom Map Projections for Regional Groundwater Models. Groundwater.

[43] Dyke AS (2004). An outline of North American deglaciation with emphasis on central and northern Canada. Dev. Quat. Sci..

[44] Dyke, A. S., Moore, A. & Robertson, L. Deglaciation of North America. *Geological Survey of Canada, Open File* **1574**, 10.4095/214399 (2003).

[45] GEBCO Compilation Group. GEBCO 2021 Grid. 10.5285/c6612cbe-50b3-0cff-e053-6c86abc09f8f (2021).

[46] Tozer B (2019). Global bathymetry and topography at 15 arc sec: SRTM15+. Earth Space Sci..

[47] Morlighem M (2017). BedMachine v3: Complete bed topography and ocean bathymetry mapping of Greenland from multibeam echo sounding combined with mass conservation. Geophys. Res. lett..

[48] Leverington DW, Teller JT, Mann JD (2002). A GIS method for reconstruction of late Quaternary landscapes from isobase data and modern topography. Comput. Geosci..

[49] Lewis MCF, Blasco SM, Gareau PL (2005). Glacial isostatic adjustment of the Laurentian Great Lakes basin: using the empirical record of strandline deformation for reconstruction of early Holocene paleo-lakes and discovery of a hydrologically closed phase. Géog. Phys. Quat..

[50] Shaw J, Gareau P, Courtney R (2002). Palaeogeography of Atlantic Canada 13–0 kyr. Quat. Sci. Rev..

[51] Oakley BA, Boothroyd JC (2012). Reconstructed topography of Southern New England prior to isostatic rebound with implications of total isostatic depression and relative sea level. Quat. Res..

[52] Godbout P-M, Brouard E, Roy M (2022). PANGAEA.

[53] ASPRS. ASPRS positional accuracy standards for digital geospatial data. *Photogramm. Eng. Remote Sens*. **81**, A1–A26, 10.14358/PERS.81.3.A1-A26 (2015).

[54] Gesch DB (2018). Best practices for elevation-based assessments of sea-level rise and coastal flooding exposure. Front. Earth Sci..

[55] Kelly KM, Dennis ML (2022). Transforming between WGS84 Realizations. J. Surv. Eng..

[56] Craymer MR (2006). The evolution of NAD83 in Canada. Geomatica.

[57] Simon KM, Riva REM (2020). Uncertainty estimation in regional models of long‐term GIA uplift and sea level change: An overview. J. Geophys. Res.: Solid Earth.

[58] Shi W, Wang B, Tian Y (2014). Accuracy analysis of digital elevation model relating to spatial resolution and terrain slope by bilinear interpolation. Math. Geosci..

[59] Simon KM, James TS, Dyke AS (2015). A new glacial isostatic adjustment model of the Innuitian Ice Sheet, Arctic Canada. Quat. Sci. Rev..

[60] Lambeck K, Rouby H, Purcell A, Sun Y, Sambridge M (2014). Sea level and global ice volumes from the Last Glacial Maximum to the Holocene. Proc. Natl. Acad. Sci. USA.

[61] Dalton AS (2020). An updated radiocarbon-based ice margin chronology for the last deglaciation of the North American Ice Sheet Complex. Quat. Sci. Rev..

[62] Naylor S, Wickert AD, Edmonds DA, Yanites BJ (2021). Landscape evolution under the southern Laurentide Ice Sheet. Sci. Adv..

[63] Wickert AD (2016). Reconstruction of North American drainage basins and river discharge since the Last Glacial Maximum. Earth Surf. Dyn..

[64] Brouard E, Roy M, Godbout P-M, Veillette JJ (2021). A framework for the timing of the final meltwater outbursts from glacial Lake Agassiz-Ojibway. Quat. Sci. Rev..

[65] Wickham, H. *ggplot2: Elegant Graphics for Data Analysis (2nd edition)*. Springer International Publishing (2016).

[66] R Core Team. R: A language and environment for statistical computing. R Foundation for Statistical Computing, Vienna, Austria. Available online at https://www.R-project.org/ (2018).

