# Publisher Correction: 1-km resolution rebound surfaces and paleotopography of glaciated North America since the Last Glacial Maximum

**DOI:** 10.1038/s41597-023-02719-6

**Published:** 2023-11-08

**Authors:** Pierre-Marc Godbout, Etienne Brouard, Martin Roy

**Affiliations:** 1 Geological Survey of Canada, Natural Resources Canada, 601 Booth Street, Ottawa, ON K1A 0E8 Canada; 2 Department of Earth and Atmospheric Sciences & GEOTOP Research Center, University of Quebec at Montreal, C.P. 8888, Succ. Centre-ville, Montreal, QC H3C 3P8 Canada

**Keywords:** Geology, Geography, Geomorphology

Correction to: *Scientific Data* 10.1038/s41597-023-02566-5, published online 23 October 2023

The copyright holder for this article was incorrectly given as ‘His Majesty the Queen in Right of Canada as represented by the Minister of Natural Resources 2023’, but should have been ‘His Majesty the King in Right of Canada as represented by the Minister of Natural Resources 2023’.

The original article has been corrected.

